# Development of a Core Outcome Set for the Benefits and Adverse Events of Acute Heart Failure in Clinical Trials of Traditional Chinese Medicine and Western Medicine: A Study Protocol

**DOI:** 10.3389/fmed.2021.677068

**Published:** 2021-05-07

**Authors:** Ruijin Qiu, Songjie Han, Xuxu Wei, Changming Zhong, Min Li, Jiayuan Hu, Pengqian Wang, Chen Zhao, Jing Chen, Hongcai Shang

**Affiliations:** ^1^Key Laboratory of Chinese Internal Medicine of Ministry of Education and Beijing, Beijing University of Chinese Medicine Affiliated Dongzhimen Hospital, Beijing, China; ^2^Beijing University of Chinese Medicine Third Affiliated Hospital, Beijing, China; ^3^Institute of Chinese Materia Medica, China Academy of Chinese Medical Sciences, Beijing, China; ^4^Institute of Basic Research in Clinical Medicine, China Academy of Chinese Medical Sciences, Beijing, China; ^5^Baokang Affiliated Hospital of Tianjin University of Traditional Chinese Medicine, Tianjin, China

**Keywords:** acute heart failure, core outcome set, integrative medicine, methodology, safety and efficacy, study protocol

## Abstract

**Aims:** To identify a minimum set of efficacy and adverse events for patients with acute heart failure (AHF) among different stakeholders in clinical trials of traditional Chinese medicine and Western medicine.

**Methods and Analysis:** First, we will develop a preliminary long list of outcomes that includes efficacy and adverse events/reactions via three steps: (i) systematic reviews of efficacy and safety outcomes for clinical trials of AHF; (ii) drugs included in the National Medical Insurance Catalog, the National Essential Medicines Catalog, and the WHO Essential Medicines List will be collected and safety outcomes extracted from the package inserts; and (iii) patients' or caregivers' semi-structured interviews will be carried out to add new viewpoints to the list. Second, after merging outcomes and grouping them under different outcome domains, questionnaires for health professionals and patients will be separately developed. Further, two rounds of Delphi survey for health professionals and a survey for patients and the public will be carried out. Third, different stakeholders will discuss and determine the final core outcome set (COS) for AHF in a consensus meeting.

**Ethics and Dissemination:** The entire project has been approved by the Ethics Committee of the main institution. After the final COS is developed, it will be published and discussed widely in conferences.

**Clinical Trial Registration:** This study is registered with the Core Outcome Measures in Effectiveness Trials database as study 1566 (available at: https://www.cometinitiative.org/Studies/Details/1566).

## Introduction

Acute heart failure (AHF) is a life-threatening condition associated with a high risk of mortality and readmission. Therapies for AHF include intravenous diuretics with adjunctive vasodilators and inotropes. However, studies have failed to prove the long-term efficacy obtained from new therapies or drugs in the past decades for patients with AHF (1, 2).

In China, traditional Chinese medicine (TCM), which is proven to be effective in protecting the myocardium, has been used to treat cardiovascular diseases, including AHF (3). It is important to merge data or compare outcomes between randomized controlled trials (RCTs) that employ different interventions. However, a large number of trials were excluded because no important outcomes were reported, and comparison and meta-analyses were hampered because of the heterogeneity of outcome reporting in systematic reviews (4–6). This could reduce the value of clinical trials and increase waste. To improve the consistency of outcomes in future clinical trials, it is necessary to develop a core outcome set (COS).

A COS is an agreed minimum set of outcomes that should be measured and reported in all clinical trials of a particular disease or health condition (7). Researchers are also encouraged to report other outcomes that are not included in a COS (8). Using a COS has potential benefits such as reducing the heterogeneity of outcomes reporting in different clinical trials as well as reducing outcome reporting bias when all clinical trials report predetermined outcomes.

The sixth annual update of a systematic review of COS showed that there were 370 published (1981–2019) COS studies documenting clinical research (9). A research assessed the uptake of COS in RCTs or systematic reviews, and the results showed that the uptake rates reported for RCTs varied from 0% RCTs (gout) to 82% RCTs (rheumatoid arthritis) (10).

There is no specific defined COS for AHF that can be used in clinical trials of pharmacotherapy (including TCM and Western medicine). After searching the Core Outcome Measures in Effectiveness Trials (COMET) database, we identified four related COS for heart failure (HF). The COS for acute heart failure syndromes (AHFS) recommended potential endpoints that should be considered in clinical trials of mechanical circulatory support devices for AHFS (COS for AHFS-MCS), which is developed in 2006 (11). The European Society of Cardiology Heart Failure Association recommended clinical outcome endpoints in HF trials in 2012 (COS for HF trials) (12). Another study represents the perspectives of a workshop held in 2014, which recommended endpoints selection that should be considered for future clinical trials of HF with preserved ejection fraction (HFpEF) in targeted therapies (COS for HFpEF) (13). To our knowledge, thus far, only one international consortium has developed a standardized outcome measurement set for patients with HF, dedicated to increase quality and value in HF care (COS for HF practice) (14).

**Figure 1:**
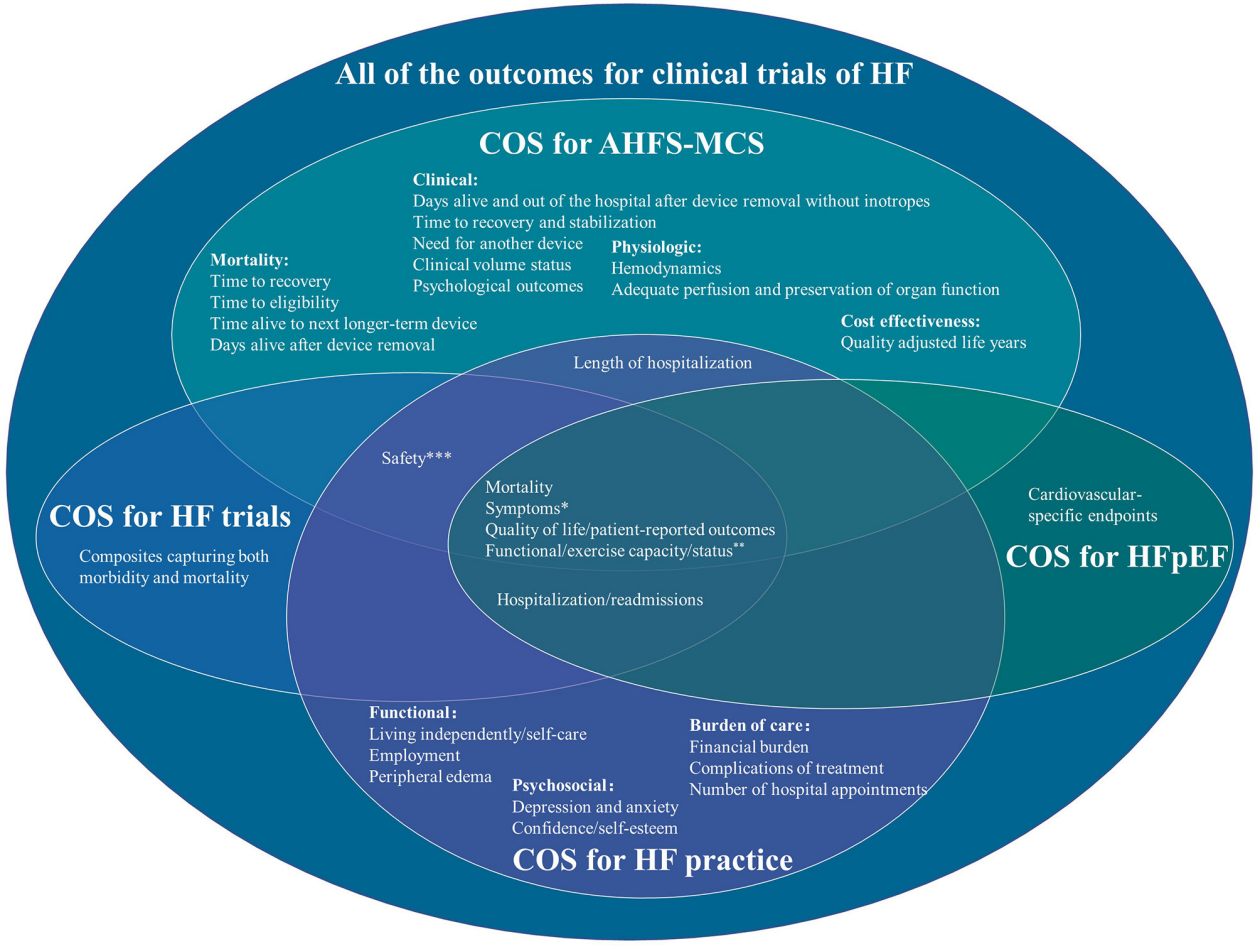
Outcomes in different core outcome sets of heart failure. *COS for AHFS-MCS: dyspnea and fatigue, COS for HF practice: shortness of breath, fatigue and tiredness, and disturbed sleep. **COS for AHFS-MCS: VO2, 6-min walk, and NYHA Class, COS for HF practice: maximum level of physical exertion. ***COS for AHFS-MCS: infection, stroke, bleeding, device failure or malfunction, procedural complications, need for device repair/removal/re-implantation. COS for HF practice: medication side-effects.

All of these related COS include stakeholders from Europe and America. Two studies include stakeholders from Asia (Singapore) and Oceania (Australia) (13, 14). The results missed the views of stakeholders from middle- and low-income areas and stakeholders that provide and accept herbs or complementary and alternative medicine. The characteristics of these related COS are shown in Table 1. The number of outcomes in different COS ranged from 6 to 24. All of these COS recommended mortality, symptoms, quality of life/patient-reported outcomes, and functional/exercise capacity/status. The outcomes of these COS are shown in Figure 1.

**Table 1:** The characteristics of related core outcome set (COS) for heart failure.

**Study ID**	**Indication**	**Intervention**	**Study method**	**Study type**	**Stakeholders**	**Geographical location**
O'Connell 2009 (11)	Acute heart failure syndromes	Mechanical circulatory support devices	-Semi–structured discussion	-COS for clinical trials or clinical research	-Clinical experts -Other (unknown)	International -Europe (France, Greece) -North America (USA)
Zannad 2013 (12)	Heart failure	/	-Semi–structured discussion	-COS for clinical trials or clinical research	-Members of a clinical trial network. -Pharmaceutical industry representatives -Regulatory agency representatives -Statisticians	International Europe (Austria, France, Germany, Greece, Italy, Norway, Poland, Spain, Sweden, Switzerland, the Netherlands, UK) -North America (Canada, USA)
Senni 2014 (13)	Heart failure with preserved ejection fraction	Targeted therapies	-Semi–structured discussion	-COS for clinical trials or clinical research	-Health professionals	International -Europe (Germany, Greece, Italy, the Netherlands, Norway, Spain, UK) -North America (USA) -Asia (Singapore) -Oceania (Australia)
Burns 2019 (14)	Heart failure	Pharmacotherapy, invasive therapies, rehabilitation	-Delphi survey -Patient focus groups -Online patient surveys -Systematic research -Teleconferences	-COS for practice	-Clinical heart failure experts Researchers Patient representatives	International -Europe (Greece, Spain, Sweden, the Netherlands, UK) -North America (USA) -South America (Brazil) -Asia (Singapore) -Oceania (Australia)

To improve the consistency of outcome reporting and reduce potential outcome reporting bias, especially for safety outcome reporting in clinical trials of AHF, and add the perspectives of stakeholders from China, we plan to develop a specific COS for AHF. In China, a large number of patients have received TCM or integrative medicine (combined TCM and Western medicine); therefore, this specific COS will be used both in clinical trials of TCM and Western medicine.

First, we will conduct systematic review, Package Inserts review, and qualitative interviews to develop a long list of outcomes (including efficacy and adverse events) for AHF. Second, we will carry out two rounds of the Delphi survey with different stakeholders to determine the importance and priority of outcomes that should be measured and reported in all clinical trials. Third, we will hold a consensus meeting to decide the final COS. This COS will be published and widely discussed in national and international conferences to encourage researchers to use it in future clinical trials of AHF. This research will be conducted in China.

The objective of this study is to identify what efficacy and adverse events should be measured in clinical trials of integrative medicine for AHF. After the COS completed, we intend to consider when and how to measure these outcomes. This protocol will be reported following the Core Outcome Set Standards for Protocol Items (COS-STAP) statement, which is shown in Supplementary Material 1 (15).

The scope of the COS is as follows:

1. Health condition: AHF, including classified as an acute first presentation or as an acute decompensation of chronic heart failure.
2. Population: patients aged between 18 and 80 years.
3. Interventions: TCM, Western medicine (pharmacotherapy) or integrated medicine.
4. Context of use: RCTs.

The study is registered in the COMET database as study 1566 (available at: http://www.comet-initiative.org/Studies/Details/1566).

## Methods and Analysis

### Steering Committee

We will form a national Steering Committee to support the development of the COS for AHF. The Steering Committee will include five experts, including two TCM/integrated medicine experts in cardiology, a Western medicine expert in cardiology, a nurse and a methodologist. The Steering Committee will review and confirm the research protocol, identify the preliminary checklist of outcomes, make decisions when there is confusion and attend the consensus meeting to facilitate COS development.

### Patient and Public Involvement

We will recruit patients and the public (caregivers and journal editors) to participate in semi-structured interviews or a questionnaire-based survey.

### Design

This COS will be developed in four phases.

Phase 1: Developing a long list of efficacy and adverse events for AHF

Phase 2: Delphi survey for health professionals

Phase 3: Survey for patients and the public

Phase 4: Consensus meeting.

The details of the process are as follows:

#### Phase 1: Developing a Long List of Efficacy and Adverse Events for AHF

There are three steps to develop a long list of efficacy and adverse events for AHF, namely: systematic reviews, Package Inserts review and semi-structured interviews.

##### Systematic Reviews

To obtain a preliminary long list of outcomes, we will conduct two systematic reviews, one is for developing a list of efficacy outcomes, and the other is for developing a list of adverse events. We will search three English databases and three Chinese databases: PubMed, the Cochrane Library, Embase, Wanfang Database, the China National Knowledge Infrastructure (CNKI) and SinoMed. Studies published from January 1, 2010 to August 1, 2020 will be retrieved. The English search strategy is presented in Supplementary Material 2. The language of publication will be restricted to English and Chinese.

The inclusion and exclusion criteria for the systematic reviews are shown in Table 2. We will extract the first author's name; number of participants; characteristics of the study; classification of HF with regard to age, sex, interventions, comparisons; and outcomes (including primary outcomes, secondary outcomes and safety outcomes); definition of outcomes/outcome measurement instruments; and outcome measurement time (intervention time/follow-up time). If TCM syndromes are reported in clinical trials of TCM/integrated medicine, TCM syndrome names and diagnostic criteria will also be extracted. In addition, we will use the MOMENT scoring system to assess the quality of outcomes reporting (16), the Risk of Bias tool to assess the quality of RCTs (17), the Newcastle-Ottawa Scale to assess the quality of cohort studies (18), and the tool developed by the Canadian Institute of Health Economics to assess the quality of case series studies (19).

**Table 2:** The inclusion and exclusion criteria of systematic reviews for reported outcomes.

**Inclusion criteria**	**Exclusion criteria**
Patients with acute heart failure	The main objectives of the study were to assess the mechanisms or pharmacokinetics of interventions
Interventions include Western medicine, TCM, and integrated medicine	Outcome measurement time or outcome definition/measurement instruments cannot be extracted
The systematic review for efficacy will include randomized controlled trials, the systematic review for safety outcomes will include any type of trials, such as randomized controlled trials, case reports and observational studies	No information on ethical approval/funding/trial registration
The clinical trials were published in Chinese or English only	Full-text cannot be obtained

Two researchers will independently extract the information and assess the quality of each study. After cross-checking, any disagreement will be resolved by discussion or via consulting with the third investigator.

##### Drug Package Inserts Review

Interventions of AHF will be extracted based on the newest clinical practice guideline. Then, two researchers will choose drugs from the National Medical Insurance Catalog, the National Essential Medicines Catalog, and the WHO Essential Medicines List. One investigator will extract adverse events/effects from drug package inserts. The other investigator will check the results. New adverse events/effects will be added to the list.

##### Semi-structured Interviews

We will recruit patients with AHF (or their caregivers) to participate in semi-structured interviews. The inclusion and exclusion criteria for the semi-structured interview are presented in Table 3. We will recruit at least 30 patients or caregivers, which we believe will achieve sufficient saturation in the semi-structured interview, that means no new ideas occur (20). Data will be analyzed upon completion of the interview. When there is a new point of view in the final interview, we will recruit more patients or caregivers to participate, until no new point of view is generated. We will recruit patients or caregivers by simple sampling.

**Table 3:** The inclusion and exclusion criteria for semi-structured interview.

**Inclusion criteria**	**Exclusion criteria**
Patients with acute first presentation of heart failure or acute decompensation of chronic heart failure	Patients with severe mental disease, cancer, and other life-threatening diseases
Patients who are 18–80 years old	
Caregivers who are taking care of patients with AHF	
Patients/caregivers who signed the informed consent forms	

We will approach potential participants in Dongzhimen Hospital, Beijing University of Chinese Medicine. An investigator experienced in qualitative research will explain the study to the patients. All patients or caregivers will have the chance to read separate written information sheets, and informed consent forms will be signed by the ones who agree to participate in the interview. Then, a semi-structured interview will be conducted by themselves or their caregivers. The investigator will collect patients' socioeconomic, demographic, and other information in the interview.

The outline of the semi-structured interviews is as follows:

1. When did you/the patient experience AHF for the first time?
2. What inconveniences have you experienced after being diagnosed with AHF/after taking care of the patient?
3. What therapies have you/the patient received because of AHF?
4. What effect do you/the patient hope to achieve after treatment?
5. What inconveniences have you/the patient experienced from the current treatment?

The results of the semi-structured interviews will be analyzed concurrently with data collection. All interviews will be transcribed verbatim and imported into qualitative analysis software. We will use framework methodology, including familiarization, for developing a thematic framework, indexing, devising thematic charts, mapping and interpreting, to further analyze the data (21). Narrative explanations of the effects of AHF and treatments on the patients' lives will be interpreted by the process of constant comparison to identify outcomes that are important to patients (22). Then, two researchers will identify whether these outcomes are new. Any inconsistency will be discussed and consensus achieved. After review by the Steering Committee, the new ones will be added to the long list of outcomes.

#### Merging Outcomes and Grouping Under Outcome Domains

After the reviews and interviews are completed, two researchers will merge the outcomes and independently group them under outcome domains. The methods of merging outcomes and grouping under different outcome domains have been used in our previous research (23):

1. English will be translated into Chinese according to the terminology formulated by the national science and technology terminology committee. If there is no relevant term, the appropriate translation will be determined by two researchers.
2. Composite outcomes will be extracted as individual outcomes.
3. The overlapping outcomes will be merged into one according to the definition of the outcomes. For example, death, death from any cause, mortality, over-all mortality, total mortality, all causes of death and all causes of mortality will be aggregated as “all-cause mortality.”
4. Those outcomes without definition or measurement instrument will be dropped.
5. The outcomes will be grouped into different outcome domains according to the taxonomy that has been developed by the COMET initiative (24).

Two researchers will cross-check the results. Any inconsistency will be further discussed by the two researchers until a consensus is reached.

#### Phase 2: Delphi Survey for Health Professionals

##### Stakeholder Selection

We will invite health professionals, such as TCM/integrated medicine experts (clinicians and researchers) in cardiology, Western medicine experts (clinicians and researchers) in cardiology, nurses, and methodologists in evidence-based medicine to participate in the two rounds of Delphi survey. The inclusion and exclusion criteria for health professionals in the Delphi survey are given in Table 4.

**Table 4:** The inclusion and exclusion criteria for health professionals in the Delphi survey.

**Inclusion criteria**	**Exclusion criteria**
Health professionals with at least a bachelor's degree	None
Health professionals who have at least 1 year of work experience	
Clinicians and nurses who work in tertiary hospitals in China	
Researchers who have participated in the design, implementation, management or statistical analysis in clinical trials of AHF, or conducted systematic reviews of AHF in the past 10 years	
There will be no restriction on the professionals' geographical area.	

The original information of the health professionals will be identified from the Department of Cardiology in Dongzhimen Hospital, Beijing University of Chinese Medicine; the membership lists of the Alliance of Chinese and Western Medicine Clinical Research, Clinical Research Method of Cardiovascular Disease of Professional Committee of Chinese Association of Integrative Medicine; and the China Research Institute of China, Information Association for Traditional Chinese Medicine and Pharmacy.

##### Sampling Strategy

There is no standard sample size calculation method in the Delphi survey in the development of COS. In previous studies, the sample size of health professionals ranged from 12 to 174 (25). In this research, we will try to invite every eligible participant from the aforementioned institutions to participate in the Delphi survey. We will encourage participants to forward the online survey to their colleagues who are eligible. The sample size for each stakeholder will be at least 15.

##### Development of Questionnaire for Round 1 of the Delphi Survey

The questionnaire for round 1 of the Delphi survey will include all candidate outcomes in different outcome domains and scoring. A nine-point scoring system will be used in the questionnaire, wherein “1–3” indicates that the outcome is not important in the COS, “4–6” indicates that the outcome is important but not critical in the COS, and “7–9” indicates that the outcome is critical in the COS (23, 26). Participants will also have the option to choose “unclear” for each outcome, if they find it difficult to score in terms of importance. At the end of the questionnaire, there will be one open-ended question: which outcomes do you think are important but are not included in the questionnaire?

##### Round 1 of the Delphi Survey

Round 1 of the Delphi survey will last for 3 weeks. The questionnaire will be sent by email or smartphone APP to potential participants. The participants will be asked to forward the questionnaire to their colleagues. We will send emails or messages to remind potential participants to complete the Delphi survey at the end of the 2nd weekend.

##### Data Analysis for Round 1 of the Delphi Survey

Data analysis for round 1 of the Delphi survey will include the frequencies of the response options for each outcome. If an outcome is scored as 7–9 by no more than 10% participants who complete the questionnaire, it will be excluded from round 2 of the Delphi survey. If participants recommend outcomes that are not included in round 1 of the Delphi survey, two researchers will identify if they are new ones. New outcomes will be included in round 2 of the Delphi survey.

##### Round 2 of the Delphi Survey

Round 2 of the Delphi survey will be sent to participants who complete round 1 of the Delphi survey. In the questionnaire, the participants will receive their score from round 1 of the Delphi survey and the score distribution of their own stakeholders. They will be asked to re-score the outcomes within 3 weeks. We will send emails or messages to remind participants to complete the Delphi survey at the end of the 2nd weekend. If the response rate is <80%, we will keep the Delphi survey open longer, or invite other eligible people to participate in the survey.

##### Data Analysis for Round 2 of the Delphi Survey

Data analysis for round 2 of the Delphi survey will include the response rate; the frequencies of the response options for each outcome from different stakeholders; the number of participants who score differently among those who complete both round 1 and round 2; the outcomes that achieve “consensus in,” “consensus out” and “no consensus;” and the potential attrition bias. The attrition bias will be calculated by the mean score in participants who complete or do not complete the two rounds of the survey. If the attrition bias is because of the participants that do not complete round 2 of the Delphi survey, we will calculate the mean score for each outcome in participants who complete or do not complete the two rounds of the survey. The statistically significant outcomes will be discussed in the consensus meeting.

The consensus definitions are as follows, which have been used in previous research (23, 27):

Consensus in (the outcome that should be included in the COS): 70% or more of the participants scored outcome as 7–9, and <15% of the participants scored the outcomes as 1–3.

Consensus out (the outcome that should not be included in the COS): 50% or less of the participants scored the outcome as 7–9.

No consensus (the importance of the outcome remains uncertain): anything else.

#### Phase 3: Survey for Patients and the Public

##### Stakeholder Selection

We will invite patients to participate in the survey for patients and the public.

The inclusion and exclusion criteria are given in Table 5.

**Table 5:** The inclusion and exclusion criteria for patients and the public survey.

**Inclusion criteria**	**Exclusion criteria**
Patients with acute first presentation of heart failure or acute decompensation of chronic heart failure	Patients with severe mental disease, cancer, and other life-threatening diseases
Patients who are 18–80 years old	Participants who cannot communicate with others
Patient caregivers should have the experience of caring for patients with AHF, including care workers and family members	Participants who cannot read and write
Journal editors should have at least 3 years' work experience	
Participants who signed the informed consent forms	

We will approach potential patients in Dongzhimen Hospital, Beijing University of Chinese Medicine. An investigator will explain the study to the patients. The patients will have chance to read separate written information sheets, and an informed consent form will be signed by the ones who agree to participate in the survey. Then, the patients will get a printed questionnaire or online questionnaire that is sent by smartphone. They can complete the questionnaire with the help of the investigator.

##### Sampling Strategy

From the previous COS studies, the number of patients ranged from 32 to 185 (25). In this research, we will recruit at least 32 patients and the public.

##### Development of Questionnaire for Patients and the Public

From the experience of the previous COS studies, we found that it is difficult for patients to score the importance of outcomes, either because they do not understand the significance of clinician-reported outcomes such as laboratory/biomarkers and outcomes that can be observed/measured by trained professionals or because they believe that all outcomes are important and should be measured.

In this research, we will develop a simple questionnaire with understandable language for patients and the public. The outcome domains will be listed in the questionnaire. If the outcome domains include individual subjective outcomes that can be observed (e.g., vomiting) or primarily observable outcomes with subjective components (e.g., nail discoloration), the outcomes will be listed under the outcome domains.

In the questionnaire for patients and the public, participants will be asked to vote on which outcomes/outcome domains are important to them and should be measured in all clinical trials. At the end of the questionnaire, there will be one open-ended question: which outcomes do you think are important but are not included in the questionnaire?

##### Data Analysis of Questionnaire for Patients and the Public

The frequencies of outcomes/outcome domains voted by each stakeholder will be calculated. If an outcome/outcome domain is voted by ≥70% participants, it will be defined as “consensus in.” If an outcome/outcome domain is voted by <50% participants, it will be defined as “consensus out.” If an outcome/outcome domain is voted by 50–70% participants, it will be defined as “no consensus.”

If there are new outcomes recommended by patients and the public, two researchers will identify if they are new ones. The new ones will be discussed if measurable. If so, they will be included in the consensus meeting.

#### Phase 4: Consensus Meeting

##### Stakeholder Selection

We will hold a face-to-face consensus meeting after analyzing the data of the surveys. The Steering Committee will be included in the consensus meeting. We will also invite different stakeholders to participate. For the health professionals, the inclusion and exclusion criteria are as listed in Table 6. For the patients and the public, we will respectively invite one participant who completes the questionnaire from each stakeholder to attend the meeting.

**Table 6:** The inclusion and exclusion criteria for health professionals in consensus meeting.

**Inclusion criteria**	**Exclusion criteria**
The health professionals should have at least a Sino Med master's degree	None
The health professionals should have more than 5 years of work experience.	
The clinicians and nurses should have work experience in tertiary hospitals.	
There will be no restriction on the professional's geographical area.	
The researchers should have participated in the design, implementation, management or statistical analysis in clinical trials of AHF, or conducted systematic reviews of AHF in the past 10 years.	
There will be no restriction on whether the health professionals participate in the Delphi survey	

##### Sampling Strategy

There is no standard sample size calculation method for the process of the consensus meeting. To obtain different stakeholders' perspectives, as well as to improve consensus achievement, we will invite at least two participants from each stakeholder to attend the consensus meeting.

##### Consensus Meeting Process

The consensus meeting will be held in China. It will last at least 1 day. In the consensus meeting, we will report the results of round 2 of the Delphi survey for health professionals and the results of the survey for patients and the public. The outcomes which are achieved “consensus out” by all stakeholders will be excluded. The outcomes which are achieved “consensus in” by all stakeholders will be sent to the Steering Committee and participants on the day before the consensus meeting.

In the consensus meeting, if the participants disagree with any outcome that achieved “consensus in” by all stakeholders to include in the COS, they will further discuss it. No consensus outcomes will be discussed one by one. Then, all of the participants in the consensus meeting will be anonymously asked to vote as “controversial” or “no consensus” outcomes reached. The ones which are voted by ≥70% participants will be included in the final COS.

The final COS will recommend efficacy and safety outcomes that should be measured by all RCTs of AHF, including different classification of HF, such as HF with reduced EF (HFrEF), HF with mid-range EF (HFmrEF), and HFpEF.

**Figure 2:**
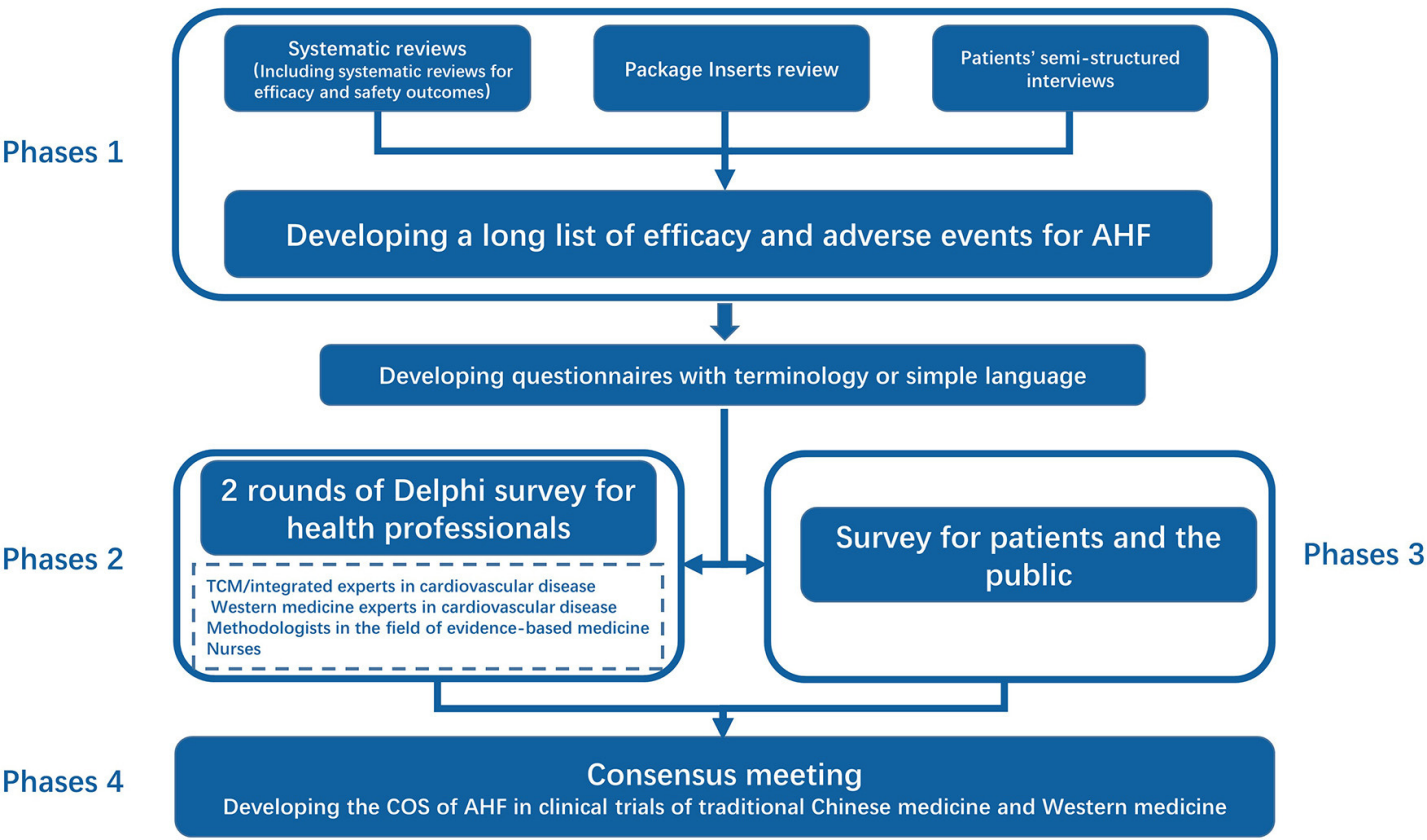
The flowchart of the study.

The flowchart of this research is shown in Figure 2.

## Discussion

At present, there are four related COS for HF (11–14). After identifying the COS, mortality, symptoms, quality of life/patient-reported outcomes, and functional/exercise capacity/status are important outcomes/outcome domains in all of the recommendations. However, there are still minor difference in individual outcomes between different COS, as well as the difference in how to measure and when to measure these outcomes. According to the scope and methods of these COS, it appears that the researchers can choose the four outcomes/outcome domains for clinical trials of AHF that are treated by pharmacotherapy when there is no specific COS.

We believe that it is necessary to develop a specific COS for AHF. There is a specific COS for mechanical circulatory support devices. This COS will only consider pharmacotherapy that include TCM and Western medicine. Only the COS for HF practice was developed by mixed methods (12), while the others were developed by qualitative research. It is unclear whether all candidate outcomes were considered in the semi-structured discussion. In this COS development, we will conduct a mixed-methods research to achieve consensus in different stakeholders.

In previous research, it is difficult to recommend safety outcomes except for surgery therapy. When the drugs for AHF are known, the adverse events/effects of these drugs could be obtained, and it is feasible to standard the adverse events/effects that should be reported in RCTs of AHF. Maybe, the adverse events/effects would not occur, and it will reduce selective reporting bias when all RCTs report common adverse events/effects.

In previous COS for HF, although different studies have recommended the same outcomes/outcome domains, the definitions, measurement instruments or measurement times are different for the same outcomes/outcome domains. These differences will also result in heterogeneity. In addition, the poor quality of the measurement properties of the measurement instruments may exaggerate or decrease the efficacy of interventions. Selecting unsuitable or poor quality of outcome measurement instruments may introduce bias and lead to a waste of resources and be unethical (28). After the final COS for AHF is completed, we will recommend one measurement instrument for one outcome on the basis of “how to select outcome measurement instruments for outcomes included in a ‘core outcome set'—a practical guideline” (29). We will also invite different stakeholders to discuss when to measure each outcome that is included in the COS.

## Ethics and Dissemination

The entire project has been approved by the Ethics Committee of Dongzhimen Hospital, Beijing University of Chinese Medicine (DZMEC-KY-2020–65). We will obtain informed consent from patients and the public who participate in the semi-structured interviews or questionnaire survey.

After the final COS is completed, we will publish this research in a journal, report the results at national and international conferences and disseminate our findings on Wechat Official Accounts Platform of China Information Association for Traditional Chinese Medicine and Pharmacy Clinical Research Information Association. We will also send the publication to researchers who participated in the Delphi surveys and the consensus meeting, so that the researchers can use it in their research.

## Author Contributions

RQ, JC, and HS drew up the research design. RQ drafted the protocol and wrote the manuscript in English. CZho, XW, and CZha participated in the design amendment and helped with project coordination. SH participated in the design of systematic review. JH, ML, and PW revised the details and the language. RQ and HS are the principal investigators of the project. All authors reviewed the manuscript content and approved the final version for submission.

## Conflict of Interest

The authors declare that the research was conducted in the absence of any commercial or financial relationships that could be construed as a potential conflict of interest.
