# Supplementary material for: Development of a Core Outcome Set for the Benefits and Adverse Events of Acute Heart Failure in Clinical Trials of Traditional Chinese Medicine and Western Medicine: A Study Protocol
Source: Front Med (Lausanne). 2021 May 7;8:677068. doi: 10.3389/fmed.2021.677068 (PMC8137966; doi:10.3389/fmed.2021.677068)
Supplement: Supplementary file 1 [file Table_1.docx]

The COS-STAP Statement

| **Domains Items** | | | **Page in the manuscript** |
| --- | --- | --- | --- |
| TITLE/ABSTRACT | | |  |
| Title | 1a | Identify in the title that the paper describes the protocol for the planned development of a COS | 1 |
| Abstract | 1b | Provide a structured abstract | 1 |
| INTRODUCTION | | |  |
| Background and objectives | 2a | Describe the background and explain the rationale for developing the COS, and identify the reasons why a COS is needed and the potential barriers to its implementation | 2 |
|  | 2b | Describe the specific objectives with reference to developing a COS | 2 |
| Scope | 3a | Describe the health condition(s) and population(s) that will be covered by the COS | 2 |
|  | 3b | Describe the intervention(s) that will be covered by the COS | 2 |
|  | 3c | Describe the context of use for which the COS is to be applied | 2 |
| METHODS | | |  |
| Stakeholders | 4 | Describe the stakeholder groups to be involved in the COS development process, the nature of and rationale for their involvement and also how the individuals will be identified; this should cover involvement both as members of the research team and as participants in the study | 3-7 |
| Information sources | 5a | Describe the information sources that will be used to identify the list of outcomes. Outline the methods or reference other protocols/papers | 3-5 |
|  | 5b | Describe how outcomes may be dropped/combined, with reasons | 5 |
| Consensus process | 6 | Describe the plans for how the consensus process will be undertaken | 5-7 |
| Consensus definition | 7a | Describe the consensus definition | 6, 7 |
|  | 7b | Describe the procedure for determining how outcomes will be added/combined/dropped from consideration during the consensus process | 6, 7 |
| ANALYSIS | | |  |
| Outcome scoring/feedback | 8 | Describe how outcomes will be scored and summarised, describe how participants will receive feedback during the consensus process | 6 |
| Missing data | 9 | Describe how missing data will be handled during the consensus process | 6 |
| ETHICS and DISSEMINATION | | |  |
| Ethics approval/informed consent | 10 | Describe any plans for obtaining research ethics committee/institutional review board approval in relation to the consensus process and describe how informed consent will be obtained (if relevant) | 5, 6, 8 |
| Dissemination | 11 | Describe any plans to communicate the results to study participants and COS users, inclusive of methods and timing of dissemination | 8 |
| ADMINISTRATIVE INFORMATION | | |  |
| Funders | 12 | Describe sources of funding, role of funders | 9 |
| Conflicts of interest | 13 | Describe any potential conflicts of interest within the study team and how they will be managed | 10 |

COS: core outcome set
