# Supplementary material for: Development of a Core Outcome Set for the Benefits and Adverse Events of Acute Heart Failure in Clinical Trials of Traditional Chinese Medicine and Western Medicine: A Study Protocol
Source: Front Med (Lausanne). 2021 May 7;8:677068. doi: 10.3389/fmed.2021.677068 (PMC8137966; doi:10.3389/fmed.2021.677068)
Supplement: Supplementary file 2 [file Table_2.DOCX]

**The search strategy for benefits are as follows:**

Pubmed 2021/1/28

#1 Search: acute heart failure[MeSH Terms]

#2 Search: ((((((((HF[Title/Abstract]) OR (heart failure[Title/Abstract])) OR (cardiac failure[Title/Abstract])) OR (left ventricular dysfunction[Title/Abstract])) OR (cardiac insufficiency[Title/Abstract])) OR (AHF[Title/Abstract])) OR (myocardial failure[Title/Abstract])) OR (heart decompensation[Title/Abstract])) AND (acute[Title/Abstract])

# 3 #1 or #2

#4 Search: random*[Title/Abstract]

#5 Search: (clinical trials as topic[MeSH Terms]) NOT ((clinical trials, phase 1[MeSH Terms]) OR (Observational studies as topic[MeSH Terms]))

#6 Search: (clinical trial[Publication Type]) NOT ((observational study[Publication Type]) OR (clinical trial, phase 1[MeSH Terms]))

#7 #4 or #5 or #6

#8 #3 and #7

("2010/01/01"[Date - Publication] : "2020/08/31"[Date - Publication])

Filters: Humans

The Cochrane Library Trials

#1 (heart failure):ti,ab,kw OR (cardiac failure):ti,ab,kw OR (left ventricular dysfunction):ti,ab,kw OR (cardiac insufficiency):ti,ab,kw OR (AHF):ti,ab,kw

#2 (Myocardial failure):ti,ab,kw OR (HF):ti,ab,kw OR (Heart decompensation):ti,ab,kw

#3 #1 OR #2

#4 (acute):ti,ab,kw

#5 #3 AND #4

#6 random*

#7 #5 AND #6

Embase

#1 'heart failure':ab,ti OR 'cardiac failure':ab,ti OR 'left ventricular dysfunction':ab,ti OR 'cardiac insufficiency':ab,ti OR ‘AHF’:ab,ti OR 'myocardial failure':ab,ti OR ‘HF’:ab,ti OR 'heart decompensation':ab,ti

#2 acute:ab,ti

#3 #1 AND #2

#4 (random* OR 'clinical trial':ab,ti)

#5 #3 AND #6 AND [2011-2020]/py

**The search strategy for safety outcomes are as follows:**

PubMed

#1 Search: acute heart failure[MeSH Terms]

#2 Search: ((((((((HF[Title/Abstract]) OR (heart failure[Title/Abstract])) OR (cardiac failure[Title/Abstract])) OR (left ventricular dysfunction[Title/Abstract])) OR (cardiac insufficiency[Title/Abstract])) OR (AHF[Title/Abstract])) OR (myocardial failure[Title/Abstract])) OR (heart decompensation[Title/Abstract])) AND (acute[Title/Abstract])

# 3 #1 or #2

#4 ((((((adverse drug reaction[Title/Abstract]) OR (adverse effect[Title/Abstract])) OR (side effect[Title/Abstract])) OR (anaphylaxis[Title/Abstract])) OR (allergic reaction[Title/Abstract])) OR (safety[Title/Abstract])) OR (toxicity[Title/Abstract])

#5 #3 and #4

The Cochrane Library Trials

#1 (heart failure):ti,ab,kw OR (cardiac failure):ti,ab,kw OR (left ventricular dysfunction):ti,ab,kw OR (cardiac insufficiency):ti,ab,kw OR (AHF):ti,ab,kw OR (Myocardial failure):ti,ab,kw OR (HF):ti,ab,kw OR (Heart decompensation):ti,ab,kw

#2 (adverse drug reaction):ti,ab,kw OR (adverse effect):ti,ab,kw OR (side effect):ti,ab,kw OR (anaphylaxis):ti,ab,kw OR (allergic reaction):ti,ab,kw OR (safety):ti,ab,kw OR (toxicity):ti,ab,kw

#3 #1 AND #2

Embase

#1 'heart failure':ab,ti OR 'cardiac failure':ab,ti OR 'left ventricular dysfunction':ab,ti OR 'cardiac insufficiency':ab,ti OR ahf:ab,ti OR 'myocardial failure':ab,ti OR hf:ab,ti OR 'heart decompensation':ab,ti

#2 acute:ab,ti

#3 #1 AND #2

#4 'adverse drug reaction':ab,ti OR 'adverse effect ':ab,ti OR 'side effect ':ab,ti OR 'anaphylaxis ':ab,ti OR ‘allergic reaction’:ab,ti OR 'safety':ab,ti OR ‘toxicity’:ab,ti

#5 #3 AND #4 AND [2011-2020]/py
